# A New Characterization of Mental Health Disorders Using Digital Behavioral Data: Evidence from Major Depressive Disorder

**DOI:** 10.3390/jcm10143109

**Published:** 2021-07-14

**Authors:** Dekel Taliaz, Daniel Souery

**Affiliations:** 1 Taliaz Ltd., Tel Aviv-Yafo 6801294, Israel; 2 EPSYLON—Psychiatric Care Network, 1000 Brussels, Belgium; dsouery@psypluriel.be; 3 Laboratory of Psychological Medicine and Addictology, Faculty of Medicine, Université Libre de Bruxelles, 1050 Brussels, Belgium

**Keywords:** digital phenotyping, digital biomarkers, personalized psychiatry, Research Domain Criteria, major depressive disorder

## Abstract

Mental health disorders are ambiguously defined and diagnosed. The established diagnosis technique, which is based on structured interviews, questionnaires and data subjectively reported by the patients themselves, leaves the mental health field behind other medical areas. We support these statements with examples from major depressive disorder (MDD). The National Institute of Mental Health (NIMH) launched the Research Domain Criteria (RDoC) project in 2009 as a new framework to investigate psychiatric pathologies from a multidisciplinary point of view. This is a good step in the right direction. Contemporary psychiatry considers mental illnesses as diseases that manifest in the mind and arise from the brain, expressed as a behavioral condition; therefore, we claim that these syndromes should be characterized primarily using behavioral characteristics. We suggest the use of smartphones and wearable devices to passively collect quantified behavioral data from patients by utilizing digital biomarkers of mental disorder symptoms. Various digital biomarkers of MDD symptoms have already been detected, and apps for collecting this longitudinal behavioral data have already been developed. This quantified data can be used to determine a patient’s diagnosis and personalized treatment, and thereby minimize the diagnosis rate of comorbidities. As there is a wide spectrum of human behavior, such a fluidic and personalized approach is essential.

## 1. Introduction

The problematic heterogeneity of mental disorders is a well-discussed issue [1,2,3]. Olbert et al. [2] used combinatorial mathematics to show that two individual patients who have the same psychiatric diagnosis may not share any symptoms. In addition, two combinations of symptoms of the same disorder will often share less than half of the symptoms [2]. In order to conduct an in-depth exploration of the problem of and the potential digital solutions to the inconsistency in the characterization of psychiatric pathologies, in this article we focus on major depressive disorder (MDD).

MDD is a highly prevalent condition, with 6% of the adult population worldwide affected each year [4]. MDD has been recognized as a major risk factor for suicide by the World Health Organization [5]. In addition, MDD is associated with other life-threatening conditions, such as stroke [6]. As MDD is of particular medical importance, it is unfortunate that experts from the psychiatric community claim that MDD is poorly defined and diagnosed [7,8]. Santor et al. [8] mapped over 280 different depression scales to measure MDD. Another study that emphasized the unwanted heterogeneity of MDD was undertaken by Zimmerman et al. [9], who found that there are 227 possible ways to meet the DSM-IV diagnostic criteria for MDD, while only 170 different combinations occur among patients.

**Figure 1:**
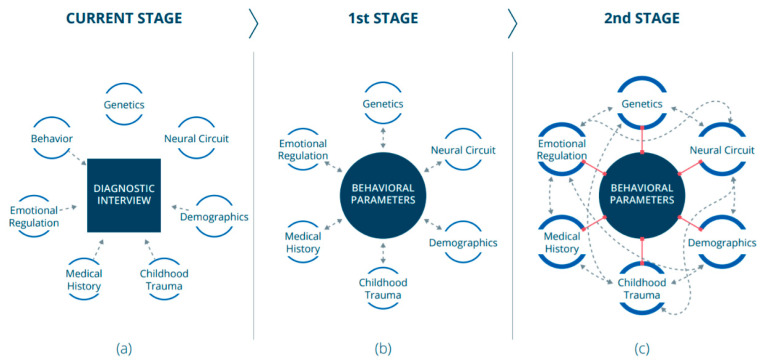
Stages toward a paradigm shift: (**a**) Today, established diagnoses of mental disorders are based on interviews. Different disciplines (smaller circles) are investigated separately. Some of those investigated areas contribute (unidirectional dashed arrows) to the diagnosis. (**b**) At the first stage, behavioral data should become the field’s pillar. Data from other relevant areas (smaller circles) will be cross-referenced (dashed bidirectional arrows) with behavioral data. (**c**) At the second stage, a substantial understanding of the behavioral component could lead to finding correlations (red lines) between behavioral markers and markers from other areas (smaller circles). This would promote crosstalk (dashed bidirectional arrows) between the different areas and consequently expand our knowledge in those areas.

This evidence indicates the problematic diagnosis system established by the Diagnostic and Statistical Manual of Mental Disorders (DSM), which is routinely used by psychiatrists. The DSM-5 diagnosis method for MDD includes a list of nine symptoms applied as diagnostic criteria [10]. Patients meet the diagnostic criteria based on the number and duration of symptoms and signs. Threshold scores are used to classify and measure depression severity. The conventional way to determine those crucial scores is to use questionnaires completed by the psychiatrist (Figure 1a). Even though psychiatrists aim to determine scores objectively, this diagnosis technique, which is mostly based on data subjectively reported by the patients themselves, leaves the mental health field behind other medical areas. One of the consequences of using this problematic diagnosis technique is also reflected in the low remission rates of MDD patients, even after they are treated with different treatment options [11,12]. Other factors that are also likely to have an influence on remission rates include patients who do not routinely take their prescribed medications. In this article, we provide examples and evidence from the literature discussing MDD, but our ideas are also relevant to other mental health disorders.

From a historical viewpoint, we can observe the conceptual changes that the classification system for mental conditions has undergone. The first DSM versions were focused on collecting statistical data [13]. In the DSM-III, there was a paradigm shift to include empirically based data with the goal of producing non-biased diagnosis criteria [13]. The DSM-5 combines etiological and neurobiological research results into the definitions of mental disorders in order to improve the diagnosis process [14,15]. This approach is consistent with the Research Domain Criteria (RDoC) project of the National Institute of Mental Health (NIMH).

The NIMH launched the RDoC project in 2009, which proposed a new framework to investigate psychiatric pathologies [16]. The project’s idea is that the integration of data from different disciplines, such as genomics, neuroimaging and the clinical field, can provide a better understanding of psychiatric pathologies. Thomas R. Insel [17] explained that today’s diagnostic systems, ICD and DSM, create a common language in psychiatry but rely on observable symptoms. This approach limits physicians from performing examinations to obtain a specific diagnosis, an option that exists in other medical fields [17]. This approach also leads physicians to routinely diagnose comorbidities within patients [18,19]. In addition, Thomas R. Insel [17] mentioned that medical diagnoses that rely only on symptoms, which are reported by the patients themselves, are not only heterogeneous and imprecise, but the subsequent treatment focuses on symptom relief and prevention.

Addressing these problems that accompany the conservative and common practice of psychiatric diagnosis requires a paradigm shift. Although it is known that various aspects (e.g., genetics, chronic medical conditions and deprivation) influence the development of a mental disorder within an individual [20], mental disorders—including MDD—are behavioral conditions. It is also known that contemporary psychiatry considers mental illnesses as diseases that manifest in the mind and arise from the brain, expressing themselves as behavioral conditions [21]. Therefore, we claim that mental syndromes should be characterized primarily using behavioral characteristics. This approach does not fully correspond with the RDoC project’s view since we argue that behavioral data should be the field’s pillar. Data from other areas, such as emotional regulation and genetics, are also essential for understanding the core of the psychiatric disorder, and must therefore be cross-referenced with behavioral data (Figure 1b). As we will emphasize in the following sections, we support the use of digital devices to collect behavioral data for the purpose of psychiatric assessment.

## 2. The Digital Revolution in Mental Health

### 2.1. An Active Digital Collection of Behavioral Data

Digitally completing a singular self-reported questionnaire is a current technique in the field [22]. Furthermore, it has been shown that smartphones and wearable devices can also be utilized for repeated self-reported questionnaires [23,24,25]. This method is called ecological momentary assessment (EMA). The advantage of this method is the frequent number of times in a short period that patients can be repetitively asked to answer diagnostic questions while in a natural setting, as opposed to periodical assessment at the clinic. Torous et al. [25] developed an app with an interface that allows users to answer the Patient Health Questionnaire-9 (PHQ-9). Suhara, Xu and Pentland [24] developed the Cognition Kit app, which asks patients to report their moods and perform digital cognitive tasks [24].

The data collected using these apps correlates with the data collected using traditional methods [23,24,25]. Although these apps show progress in the data collection method in MDD patients, they are based on conventional methods of symptom-based self-reported diagnosis.

### 2.2. Digital Phenotyping

To perform the characterization of MDD via behavioral features, patient behavior needs to be quantified objectively. At present, patients self-report about their own behavior. Physicians try their best to objectively fill the diagnosis questionnaires and determine the quantified scores. Nevertheless, the existing data are still biased. In addition, the reported behavioral data are not longitudinal and are collected during meetings between patients and their physicians. Most of the time, this is based on retrospective recollection. To overcome these limitations, we suggest the use of smartphones and wearable devices to collect quantified behavioral data from patients passively. This process falls under the definition of digital phenotyping, as defined in 2016 by Onnela and Rauch [26]. The utilization of smartphones and wearable devices exists in many other medical areas. For example, the monitoring of glucose levels among diabetes patients was revolutionized by developing and producing low-cost continuous glucose monitoring sensors [27]. Recently, decision-making using data recorded by these devices was approved by the FDA [27]. Another example of data collection via wearable devices is that done with the Apple Watch or other fitness bands that can passively measure pulse rate and detect pulse irregularity, which can signal atrial fibrillation or flutter [28]. Embracing smartphones and wearable devices as sensors for collecting quantified behavioral data could generate a quantified, continuous and objective database of patients’ behavior. Applying a computational algorithm to this database could enable different mental disorders to be subtyped into subcategories using data-driven analysis and combined with other relevant dimensions. This computational system could help determine better disease diagnoses and treatments for new patients. Of course, there are limitations to this digital approach. Since personal data about patients are collected in face-to-face meetings, unique nuances can be overlooked; a professional clinician has the potential to detect information that a machine would miss.

Torous, Onnela and Keshavan [29] previously suggested the use of digital phenotyping to collect data as part of the RDoC project. Our idea is novel in that we suggest that digital-behavioral biomarkers can not only assist in the diagnosis of MDD and other mental disorders, but that a new characterization of subtypes of such disorders should be based on them.

Our vision of embracing smartphones and wearable devices to collect data among patients suffering from mental health disorders is very reasonable since the use of these devices is growing tremendously, and 50% of all smartphones and tablets have a mobile health app downloaded [30].

The digital phenotyping field is growing. In 2018, it was suggested that actions recorded by smartphones, such as typing and scrolling patterns, can reflect the results of tests conducted by psychiatrists to assess mental health patients [31]. Potential behavioral biomarkers can be researched, relying on the information available in the DSM and ICD. Later, new biomarkers can be detected through the new data that will be gathered. Several studies performed in recent years have already produced promising results regarding digital biomarkers that can be used for MDD characterization and diagnosis. These biomarkers include the utilization of a variety of data types to detect depressive behavior. Mundt et al. and Zhang et al. [32,33,34] were able to extract features from voice samples and use them to measure depression symptoms. Actigraphy was used to measure patterns of motor activity and was employed for the development of digital biomarkers [35,36,37]. Tonon et al. [37] were able to use light exposure measurements to differentiate between melancholic depression and non-melancholic depression patients. Jacobson et al. [36] were able to draw associations between light exposure and depression severity. Saeb et al. [38] found a correlation between the severity of depression symptoms and a number of features that were extracted from mobile phone global positioning systems (GPS) and smartphones’ normal usage (usage duration and frequency). Dagum [39] analyzed the human–computer interaction of normal smartphone usage to identify digital biomarkers associated with cognitive function. Mandryk and Birk [40] conducted a literature review and suggested five categories of potential biomarkers that can be deduced from data recorded from individuals playing computer games.

Several apps for the passive collection of behavioral data via smartphones have already been developed [40]. The Mobilyze! app, developed in 2011, includes machine learning models for predicting patients’ moods [41]. Marzano et al. [42] developed a prototype system app that was tested in a small trial and was shown to accumulate both quantitative and qualitative data. Beiwe is a research platform that can passively collect various behavioral data types from users’ smartphones and transfer this data to another server for analysis. This app is currently only available for use by a small group of researchers [43].

A designated algorithm for analyzing the collected data is necessary for further development. Lydon-Staley et al. [44] created a computational algorithm that applied network science methodologies to a longitudinal behavioral database to learn about interactions between psychiatric symptoms. As a result, Lydon-Staley et al. [44] offered a robust framework to capture dynamic symptom networks.

The link between emotions and emotion regulation in psychopathology has been demonstrated in the past [41]. The transition between different emotional states and moods is substantial in psychopathology research. Therefore, digital biomarkers for emotional changes are essential. Research by Pratap et al. [43] found that mobility and smartphones’ normal usage has the potential to predict an individual’s mood state changes using personalized models. Further important work was published recently by Sultana, Al-Jefri and Lee [45], in which machine learning algorithms were used to analyze varied data recorded from individuals’ smartphones and smartwatches to determine emotional states and transitions.

Combining omics data together with the collected behavioral data could be another essential strategy to achieve better disease diagnoses. As genetic and proteomic biomarkers for MDD are routinely revealed [42,46,47], this option is becoming a reality. Olmert et al. [22] launched the Delta Trial, where blood spot samples and psychiatric digital questionnaire answers were collected from patients. Both proteomic biomarkers and symptomatic behavioral data were collected digitally and used to differentiate between bipolar disorder and MDD patients [22].

## 3. Toward Personalized Psychiatry

Since MDD is only one example among many poorly defined and diagnosed mental disorders, an innovative and fluidic approach to all mental health is necessary. Naming a set of symptoms reported by a patient does not yield satisfying outcomes. There is a wide spectrum of human behavior that cannot be expressed in one or two pathologies. The standard diagnosis of comorbidities is evidence of this incorrect classification of mental health disorders. Though personalized medicine exists in other fields [48,49,50], psychiatry has been left behind. However, innovative personal psychiatry tools for the treatment of MDD do exist [51,52,53,54]. Predictix is a decision-support tool that calculates the likely effectiveness of several antidepressant medications for individual patients, based on environmental and genetic input [54]. In an article that was accepted for publication recently, Taliaz et al. [55] successfully used data from the Sequenced Treatment Alternatives to Relieve Depression (STAR*D) study to analyze the response patterns of patients to antidepressant medications using machine learning algorithms. Expanding the applied database of such a tool with data of different mental pathologies could allow the optimum treatment to be predicted according to an individual’s specific parameters instead of prescribing medications per diagnosed condition. Furthermore, diagnoses will be shifted depending on the training and the background of the doctor, as was recently argued by Perugi and Barbuti [19].

Analysis of longitudinal behavior allows for a better comparison between a patient’s behavior in the present and their behavior in the past, as well as in a demographic context. Sleep disorders demonstrate the importance of this feature. Insomnia is one of the most common symptoms of MDD [56]. However, many studies have shown that sleep quantity and quality differ among people of different ages [57,58,59,60]. Therefore, an integration of this demographic information is crucial for an accurate diagnosis. A pilot clinical study to ascertain the feasibility of this approach is recommended.

In the future, we also believe that behavioral data could provide a reference for identifying biomarkers from other fields. A thorough understanding of the behavioral characteristics of mental disorders has the potential to find correlations between behavioral features and features from other areas (Figure 1c) and promote crosstalk between the different areas, which would result in further expanding our knowledge in those areas (Figure 1c).

As we have shown in this article, technologically, we already have the knowledge to collect the necessary behavioral data in order to learn precisely about individuals’ behavior. Therefore, personalized psychiatry should be pursued and become a standard soon.

## 4. Conclusions

Mental disorders are ambiguously defined. The currently available methods used to diagnose mental disorders are limited, as they rely on patients’ subjective self-reports. In this article, we supported these statements with examples from MDD. We suggest the use of smartphones and wearable devices to collect longitudinal data of patients’ behavior passively. Several designated apps to collect this type of data have already been developed, and digital biomarkers of MDD symptoms have already been identified. Algorithms and computational methods for analyzing continuous behavioral data have also been published. We believe all this gathered available knowledge should be integrated into one tool that can assist in diagnosing mental health patients and help bring a much-needed paradigm shift in psychiatric treatment approaches. With a better understanding of behavioral data, an integrated tool may have the potential to identify new characteristics of disorder subtypes and promote crosstalk, expanding our knowledge between and within other fields. Furthermore, quantified longitudinal behavioral data can be used to determine a patient’s diagnosis and to personalize treatment over time. This may help minimize the incorrect classification of mental health disorders and the associated diagnoses of comorbidities. Of course, rules and restrictions to preserve patients’ privacy must be established. Slavich et al. [61] suggested some guidelines for the field of speech analysis that can be generalized to other data types as well. These guidelines include informing patients of precisely what data are collected using their wearable device and enabling patients to easily stop the recording action of their device [61].

We support the RDoC framework to investigate psychiatric pathologies based on integrating biological and behavioral data from various disciplines. However, as there is a wide spectrum of human behavior that cannot be expressed in one or two pathologies, today’s medical diagnoses (that rely mainly on observable symptoms) are limited. For this reason, we believe that mental syndromes should be characterized primarily using behavioral characteristics, and that behavioral data should be the field’s pillar.
